# Neutrophil-to-Lymphocyte Ratio (NLR) and Monocyte-to-Lymphocyte Ratio (MLR) Predict Clinical Outcome in Patients with Stage IIB Cervical Cancer

**DOI:** 10.1155/2021/2939162

**Published:** 2021-09-08

**Authors:** Yong-Xia Li, Jian-Ying Chang, Ming-Yuan He, He-Ran Wang, Dai-Qin Luo, Feng-Hu Li, Jie-Hui Li, Li Ran

**Affiliations:** ^1^Department of Oncology, The Affiliated Hospital of Guizhou Medical University, Guiyang, Guizhou Province, China; ^2^Department of Breast Oncology, Guizhou Cancer Hospital, Guiyang, Guizhou Province, China; ^3^Teaching and Research Section of Oncology, Guizhou Medical University, Guiyang, Guizhou Province, China

## Abstract

**Introduction:**

Stage IIB cervical cancer (CC) is an advanced stage CC with poor prognosis. Inflammatory response plays a crucial role in the development of CC, and systemic inflammatory indexes were related to the prognosis in several cancers. The objective of the study was to determine the prognostic value of platelet-to-lymphocyte ratio (PLR), neutrophil-to-lymphocyte ratio (NLR), monocyte-to-lymphocyte ratio (MLR), basophil-to-lymphocyte ratio (BLR), and systemic inflammation response index (SIRI) as inflammatory indexes in patients with stage IIB CC.

**Materials and Methods:**

A retrospective study was performed in 260 patients with stage IIB CC. PLR, NLR, MLR, BLR, and SIRI were obtained from routine blood tests. Prognosis information of the patients was acquired from regular clinical follow-up. Recurrence and response to therapy were determined through electronic medical records (EMRs). Correlations of the inflammatory indexes with overall survival (OS), progression-free survival (PFS), recurrence, and response to therapy were analyzed using SPSS version 26.0 software.

**Results:**

Receiver operating characteristic (ROC) curve analyses suggested that NLR, MLR, and SIRI had better predictive value than PLR as well as BLR in the prognosis and recurrence risk. Both univariate and multivariate survival analyses showed that higher NLR and MLR were significantly associated with shorter OS as well as PFS, whereas SIRI was not an independent predictive factor of PFS. Chi-square test results revealed that increased NLR was significantly correlated with higher recurrence rate (*P*=0.046), and increased MLR showed significant correlation with elevated recurrence risk (*P*=0.002). Univariate and binary logistic regression analyses for response to therapy indicated that elevated NLR was associated with decreased complete remission (CR) rate (*P*=0.031), and the *P* value lost statistical significance while being adjusted by tumor size (*P*=0.108).

**Conclusions:**

For patients with stage IIB CC, both NLR and MLR are independent prognostic factors as well as risk factors for recurrence; NLR serves as a potential marker for therapeutic response.

## 1. Introduction

Cervical cancer (CC) is one of the most common female cancers, with the high mortality among women suffering from cancers, especially in developing countries [1]. Factually, CC was reported to be the fourth most frequently diagnosed cancer with approximately 527,600 newly diagnosed cases annually, and the fourth leading cause of cancer death with about 265,700 deaths each year [1]. Most of CC deaths occur in developing countries. In India, the CC deaths account for 25% of the worldwide CC deaths [2]. In China, there are about 98,900 newly diagnosed CC patients and 30,500 deaths from the cancer annually, and the incidence and mortality of CC are at the peak among female cancers [3]. A large number of CC patients still have poor prognosis despite the fact that many advances have occurred in the therapy of CC [4, 5]. Several prognosis factors are used to predict the survival of CC patients, and the patients with poor prognosis will receive more intensive chemotherapy or adjustment in the chemotherapy regimens; nevertheless, the prediction of CC prognosis is still mainly dependent on the clinical examination and imaging [6, 7]. Hence, it is meaningful to exploit more helpful and practical prognostic factors to provide a guidance in the therapy of CC, and a free or convenient access to the data of prognostic factors is necessary in the clinical practice.

Immune cells mediate inflammation response by the release of inflammatory factors to block pathological processes, probably leading to tissue injury [8, 9]. Inflammatory factors can activate immune system to promote the viability and proliferation of some malignant tumor cells, such as colorectal cancer cells. Cytokines, as inflammatory factors, are involved in the migration and motility of tumor cells and contribute to enhance the invasive ability of the tumor cells. In breast cancer, colony-stimulating factor 1 (CSF-1) was identified to promote metastatic potential leading to progression of the tumor to malignancy, and overexpression of CSF-1 is associated with poor prognosis [10, 11]. Moreover, inflammatory factors have the potential to be prognostic factors in colorectal cancer, and the risk of the mortality may be roughly evaluated by determining the plasma levels of inflammatory factors [12]. Therefore, systemic inflammatory factors, such as platelet-to-lymphocyte ratio (PLR), neutrophil-to-lymphocyte ratio (NLR), monocyte-to-lymphocyte ratio (MLR), and basophil-to-lymphocyte ratio (BLR), have been increasingly studied on the connection with cancer prognosis [13–17].

The platelet, lymphocyte, and neutrophil are important components in the tumor cell-containing microenvironment, of which the platelet promotes tumor growth and metastasis [18, 19]; the lymphocyte plays a crucial role in immunological response contributing to tumor defense [20]; the neutrophil, as the first responder to inflammation, has been increasingly recognized for involving in tumor progression and cancer development [21]. In recent years, PLR and NLR have been reported to be associated with poor prognosis in several cancers, such as hepatocellular carcinoma [22], colorectal cancer [23], and gastric cancer [24], esophageal squamous cell carcinoma [25], breast cancer [26], etc. Additionally, MLR served as a prognostic factor in patients with cancers involving colorectal cancer [27], pancreatic neuroendocrine tumors [28], gallbladder cancer [29], gastrointestinal stromal tumors [30], etc. Moreover, Prabawa IPY et al. [17] found that BLR was a risk factor for invasive cervical cancer. With respect to systemic inflammation response index (SIRI), its prognostic value was certified in several types of cancers [31–34]. Unlike genetic screening, the values of PLR, NLR, MLR, BLR, and SIRI are extremely easy to be obtained from blood routine examinations, without extra charge.

Stage IIB CC defined that the CC had invaded the parametrium, but not into the pelvic sidewall [7]. Compared to early-stage CC defined through stages IA to IIA1 with tumor size <4 cm [35], stage IIB CC had lower five-year survival rate. Moreover, the recurrence rate of stage IIB CC was high, and lymph node metastasis occurred with a high frequency in the stage IIB CC [36]. However, the data on the prognosis of stage IIB CC have been limited so far.

Although there were numerous published studies that had demonstrated the prognostic value of inflammatory indexes (PLR, NLR, MLR, BLR, and SIRI) for CC patients [17, 37–43], whether the inflammatory indexes serve as predictive factors for prognosis, recurrence, and therapeutic response in patients with stage IIB cervical cancer remains unknown. The aim of the study is to investigate the inflammatory indexes including PLR, BLR, NLR, MLR, and SIRI as the biomarkers in predicting clinical outcome in patients with stage IIB CC.

## 2. Materials and Methods

### 2.1. Patients

**Figure 1:**
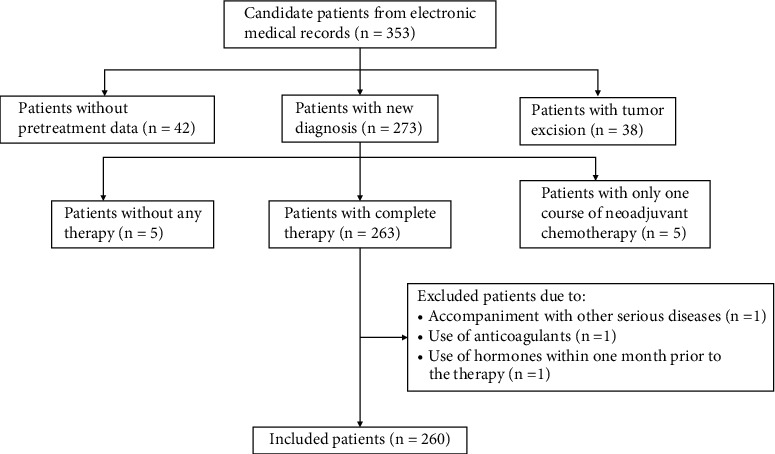
The flow chart for screening patients with stage IIB cervical cancer in the retrospective study.

The retrospective study involved 260 patients diagnosed with stage IIb cervical cancer from March 2011 to October 2016 in the Guizhou Cancer Hospital, Guiyang, Guizhou Province, China. The demographic, hematological, and pathological data of the patients were obtained from electronic medical records (EMRs), and the prognosis data were acquired from regular clinical follow-up. The staging of CC was determined by International Federation of Gynecology and Obstetrics (FIGO) stage classification (2009) involving stages I, II, III, and IV, and only stage IIb CC patients were admitted in the study. The inclusion criteria were that patients with stage IIb CC received complete therapy (neoadjuvant chemotherapy plus radiotherapy, or complete neoadjuvant chemotherapy) and underwent routine blood tests before the therapy. The exclusion criteria were as follows: (1) Use of drugs influencing routine blood tests, such as glucocorticoid, sex hormone, G-CSF (granulocyte colony-stimulating factor), interleukin, heparin, etc. (2) Accompaniment with diseases affecting peripheral blood parameters, including liver and kidney disease, myocardial infarction, connective tissue disease, and hematological disease. (3) Blood transfusion within one week prior to the therapy. 353 candidate patients with stage IIb CC were selected from EMRs in the hospital; finally, 260 patients with stage IIb CC were included in accordance with the inclusion and exclusion criteria (Figure 1).

### 2.2. Data Collection

The demographic data consisted of age, menopause, age at menopause; the hematological data referred to complete blood counts (CBCs) prior to any therapy and included white blood cell (WBC), neutrophil, lymphocyte, monocyte, red blood cell (RBC), and platelet counts, along with the hemoglobin (Hb) level; the pathological data involved histopathological classification, tumor size, and lymphatic metastasis. PLR, NLR, MLR, and BLR were calculated as the ratio of platelet count to lymphocyte count, neutrophil count to lymphocyte count, monocyte count to lymphocyte ratio, and basophil count to lymphocyte count, respectively. In addition, SIRI was determined as neutrophil count × monocyte count/lymphocyte count.

The overall survival (OS) was calculated from the date of CC diagnosis to the date of death from any cause. Progression-free survival (PFS) was determined as the time interval from the date of diagnosis to the date of clinically proven disease progression; generally, recurrence indicated disease progression. Provided that no recurrence occurred, PFS was calculated as the time interval from the date of diagnosis to the date of death. If no outcome event (recurrence or death) emerged until the end of the last follow-up, the endpoint was censored at the date of last follow-up. After several cycles of follow-up, few patients lost to follow-up, and the dates of last follow-up before losing touch were used as the censoring dates. Recurrence consisted of primary recurrence, distant metastasis, and primary recurrence plus distant metastasis. Time to recurrence was calculated from the date of CC diagnosis to the date of CC recurrence. Recurrence was the only endpoint event, and the date of death without recurrence was used as the censoring date. Five-year recurrence rate was calculated as a cumulative incidence of recurrence by the end of the five years, which was determined using statistical incidence estimates. Recurrence and therapeutic response were evaluated according to EMRs and follow-up.

### 2.3. Statistical Analysis

The relationship between inflammatory indexes (PLR, NLR, MLR, BLR, and SIRI) and clinical characteristics of patients with stage IIB cervical cancer was determined using independent Mann–Whitney *U* test. The receiver operating characteristic (ROC) curve for therapeutic response and the ROC curves for OS and PFS along with recurrence (five-year recurrence rate) were plotted using R (version 4.1.0) analysis package pROC_1.17.0.1 and Time ROC_0.4, respectively. Survival curves were plotted by a means of Kaplan–Meier method, and intergroup comparisons were performed using the log-rank tests. Cox regression analyses were used for univariate and multivariate survival analyses, and Cox proportional hazard models were used for calculating the hazard ratios (HRs). To select the variables significantly influencing CC prognosis for multivariate analysis, several common prognostic factors were screened using forward stepwise regression. Correlation between tumor size and lymphatic metastasis was determined by Chi-square test. Kaplan–Meier analysis was used to determine the cumulative risk of recurrence by the end of five years as previously described [44–48]. Association of the inflammatory indexes with the recurrence rate was determined using Chi-square test; univariate and binary logistic regression analyses were performed to determine the association of the inflammatory indexes with complete remission (CR) rate, and the association was evaluated by odds ratio (OR). All of the data analyses were performed using SPSS version 26.0 (SPSS, Inc., Chicago, IL, USA), and the difference was statistically significant when *P* < 0.05.

## 3. Results

### 3.1. Patient Characteristics

The baseline characteristics of 260 patients with stage IIB CC are described in Table 1. The median age at diagnosis of the CC patients was 51 (range 28–74) years old. Among the CC patients, 137 (52.7%) patients had gone through menopause, and the median age at menopause was 49 (range 24–57) years old. Most of the patients were classified as squamous cell carcinoma (SCC, 94.2%), of which moderately differentiated squamous cell carcinoma (WDSCC) was most common pathology. 182 (70.0%) patients were detected with tumor size of ≥4 cm; 61 (23.5%) patients underwent lymphatic metastasis. The median PLR, NLR, MLR, BLR, and SIRI of the patients were 154.17 (range 48.48–500.00), 2.49 (range 0.93–14.79), 0.26 (range 0.04–1.42), 0.021 (range 0.004–0.118), and 1.02 (range 0.04–15.39), respectively. The other hematological data are also shown in Table 1. After diagnosis of stage IIB CC, 243 (93.5%) patients received the neoadjuvant chemotherapy, of whom 72 and 123 received paclitaxel (liposome) plus cis-platinum and paclitaxel (liposome) plus lobaplatin, respectively. 256 (98.5%) patients received the radiotherapy, and the median radiotherapy dose was 56.35 (range 25.8–65.35) Gy; the median radiotherapy duration was 54 (10–96) days. 168 (64.6%) patients, 82 (31.5%) patients, 7 (2.7%) patients, and 2 (0.8%) patients achieved complete remission (CR), partial remission (PR), stable disease (SD), and progressive disease (PD) after therapy, respectively. 70 (26.9%) patients underwent cancer recurrence until the end of the last follow-up. Moreover, there were 75 (28.8%) deaths in total during the follow-up period, and 7 (2.7%) patients were lost after several follow-up. The median (range) and mean (standard deviation) follow-up periods for survival analyses were 47.3 (2.63, 101.40) months and 48.4 (23.3) months, respectively.

### 3.2. Relationship between Inflammatory Indexes and Clinical Characteristics of Patients with Stage IIB Cervical Cancer

The association of PLR, NLR, MLR, BLR, and SIRI with clinical characteristics in patients with stage IIB cervical cancer was analyzed using Mann–Whitney *U* test, in light of abnormal distribution of the values of PLR, NLR, MLR, BLR, and SIRI (Table 2). Age negatively correlated with PLR, NLR, MLR, and SIRI. Menopause was associated with lower PLR, NLR, MLR, and SIRI. The patients with SCC pathology had significantly higher NLR (*P*=0.026) and trended toward elevated MLR (*P*=0.098) and SIRI (*P*=0.072), compared to the patients with adenocarcinoma. Tumor size positively correlated with PLR, NLR, MLR and SIRI. Lymphatic metastasis was related to higher PLR, NLR, MLR, and SIRI. Neoadjuvant chemotherapy had no significant correlation with PLR, NLR, MLR, BLR, and SIRI. Obviously, BLR showed no correlation with these clinical characteristics.

### 3.3. Receiver Operating Characteristic (ROC) Curves for OS, PFS, Recurrence, and CR Rate

**Figure 2:**
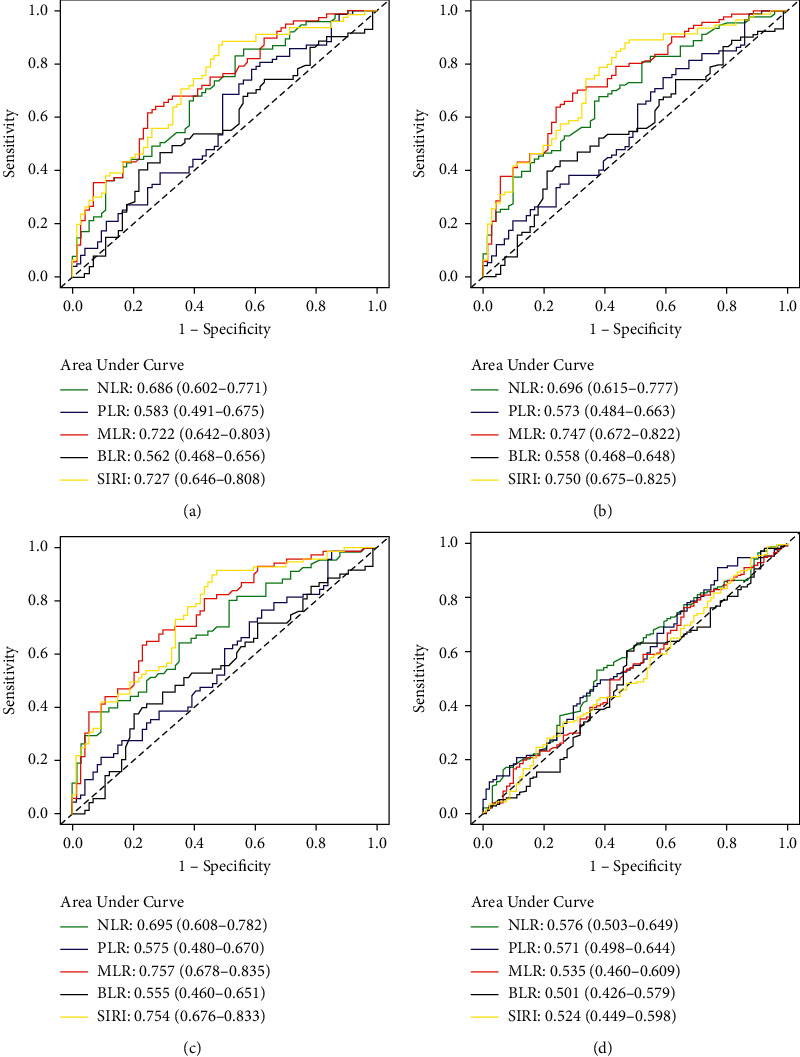
Evaluation of inflammatory indexes as predictors using receiver operating characteristic (ROC) curves in stage IIB cervical cancer. (a) Overall survival. (b) Progression-free survival. (c) Recurrence. (d) Complete remission rate.

ROC curves were performed to evaluate the predictive ability of the inflammatory indexes for OS, PFS, recurrence (five-year recurrence rate), and CR rate in patients with stage IIB cervical cancer. The predictive ability was indicated by the area under the curve (AUC). For the OS, the AUCs for PLR, NLR, MLR, BLR, and SIRI were 0.583, 0.686, 0.722, 0.562, and 0.727, suggesting that NLR, MLR, and SIRI had better predictive value than PLR as well as BLR (Figure 2(a)). With the PFS, the corresponding AUCs were 0.573, 0.696, 0.747, 0.558, and 0.750, implying that NLR, MLR, and SIRI had higher predictive value compared to PLR as well as BLR (Figure 2(b)). With respect to the recurrence, the corresponding AUCs were 0.575, 0.695, 0.757, 0.555, and 0.754, hinting that NLR, MLR, and SIRI had stronger predictive abilities compared with PLR as well as BLR (Figure 2(c)). As to the CR rate, all of the inflammatory indexes appeared to have poor predictive value, despite the fact that NLR has the largest AUC (0.576) among the inflammatory indexes (Figure 2(d)).

### 3.4. Correlation between Clinical Factors and Prognosis of Patients with Stage IIB Cervical Cancer

To identify the potential factors influencing the prognosis of patients with stage IIB CC, several clinical factors were selected, including age, menopause, pathology, tumor size, lymphatic metastasis, and neoadjuvant chemotherapy (Tables 3 and 4). Univariate OS analyses showed that the risk factors consisted of tumor size (HR = 2.130; 95% CI, 1.191–3.809; *P* = 0.011) and lymphatic metastasis (HR = 2.110; 95% CI, 1.310–3.400; *P* = 0.002). Moreover, menopause was marginally associated with better OS (HR = 0.653; 95% CI, 0.414–1.031; *P* = 0.067). Multivariate OS analyses showed that only lymphatic metastasis was the independent prognostic factor (HR = 1.806; 95% CI, 1.107–2.948; *P* = 0.018). Univariate PFS analyses revealed that only lymphatic metastasis was a significantly poor prognostic factor (HR = 1.888; 95% CI, 1.220–2.923; *P* = 0.004). Furthermore, menopause was marginally associated with longer PFS (HR = 0.687; 95% CI, 0.456–1.037; *P* = 0.074), and tumor size ≥4 cm had a trend to shorten PFS (HR = 1.514; 95% CI, 0.938–2.445; *P* = 0.09). Multivariate PFS analyses showed that only lymphatic metastasis was the independent prognostic factor (HR = 1.736; 95% CI, 1.107–2.723; *P* = 0.016). Chi-square test was performed to evaluate the correlation between tumor size and lymphatic metastasis (Table S1), and the result indicated that larger tumor size was significantly associated with lymphatic metastasis (OR = 3.60; 95% CI, 1.62–8.00; *P* = 0.001).

### 3.5. Influences of PLR, NLR, MLR, BLR, and SIRI on the Prognosis of Patients with Stage IIB Cervical Cancer

**Figure 3:**
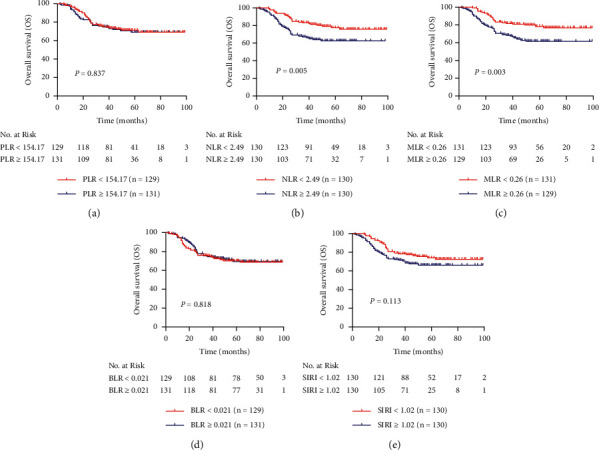
Kaplan–Meier curves for overall survival of patients with stage IIB cervical cancer in different subgroups of (a) PLR, (b) NLR, (c) MLR, (d) BLR, and (e) SIRI.

**Figure 4:**
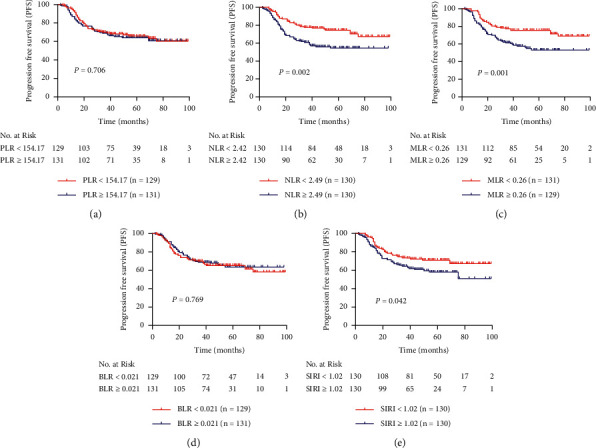
Comparison of progression-free survival of patients with stage IIB cervical cancer between different subgroups of (a) PLR, (b) NLR, (c) MLR, (d) BLR, and (e) SIRI.

The Kaplan–Meier curves of OS and PFS are shown in Figures 3 and 4, respectively. Univariate and multivariate analyses were performed to determine whether PLR, NLR, MLR, BLR, and SIRI were independent prognostic factors of OS as well as PFS in stage IIB CC patients. The values of PLR, NLR, MLR, BLR, and SIRI were cut off by corresponding median values. For all patients included in the study, the mean ± SE of OS and PFS was 75.90 ± 2.26 (95% CI 71.46–80.33) months and 68.48 ± 2.47 (95% CI 64.91–74.58) months, respectively. As shown in Table 5, higher NLR was associated with shorter OS (HR = 1.960; 95% CI, 1.227–3.131; *P* = 0.005) and PFS (HR = 1.944; 95% CI, 1.274–2.967; *P* = 0.002). After adjustment with lymphatic metastasis, higher NLR was still associated with worse OS (adjusted HR = 1.721; 95% CI, 1.068–2.773; *P* = 0.026) and PFS (adjusted HR = 1.736; 95% CI, 1.129–2.669; *P* = 0.012). MLR ≥ 0.26 was associated with shorter OS (HR = 2.012; 95% CI, 1.258–3.217; *P* = 0.003) and PFS (HR = 1.992; 95% CI, 1.303–3.043; *P* = 0.001), and when adjusted by lymphatic metastasis, MLR ≥ 0.26 still predicted worse OS (HR = 1.780; 95% CI, 1.099–2.881; *P* = 0.019) and PFS (HR = 1.806; 95% CI, 1.169–2.791; *P* = 0.008). Increased SIRI was significantly correlated with shorter PFS (HR = 1.538; 95% CI, 1.015–2.329; *P* = 0.042), but not OS (HR = 1.446; 95% CI, 0.916–2.283; *P* = 0.113). Whereas it was adjusted by lymphatic metastasis, increased SIRI was not any longer associated with shorter PFS (adjusted HR = 1.379; 95% CI, 0.901–2.110; *P* = 0.139). Neither PLR nor BLR was not a prognostic factor in stage IIB CC patients.

### 3.6. Association of Inflammatory Indexes with Recurrence and Response to Therapy of Patients with Stage IIB Cervical Cancer

Chi-square test was performed to determine the relationship between the clinical factors and recurrence in patients with stage IIB CC. The results showed that lymphatic metastasis was significantly associated with higher recurrence risk (39.2% vs. 25.2%; OR = 1.946; 95% CI, 1.060–3.571; *P*=0.030.  Table S2). As shown in Table 6, NLR ≥ 2.49 was significantly correlated with higher recurrence rate (30.5% vs. 19.8%; OR = 1.704; 95% CI, 0.913–3.185; *P*=0.046), and MLR ≥ 0.26 showed significant correlation with increased recurrence risk (37.2% vs. 19.8%; OR = 2.392; 95% CI, 1.370–4.184; *P*=0.002). There was no association of PLR, BLR, and SIRI with recurrence risk in patients with stage IIB CC.

Univariate and binary logistic regression analyses were performed to determine the association of clinical factors with CR rate in stage IIB cervical cancer (Table S3). Menopause was associated with improved CR rate (70.6% vs. 58.5%; OR = 0.798; 95% CI, 0.461–1.381; *P* = 0.042). Tumor size ≥4 cm was associated with lower CR rate (59.3% vs. 77.9%; OR = 2.415; 95% CI, 1.309–4.464; *P* = 0.004). Increased age was marginally associated with improved CR rate (70.8% vs. 59.7%; OR = 0.610; 95% CI, 0.363–1.026; *P* = 0.062). Moreover, lymphatic metastasis trended toward decreased CR rate (55.7% vs. 67.2%; OR = 1.626; 95% CI, 0.903–2.924; *P* = 0.103). Binary logistic regression analysis showed that only tumor size was an independently predictive factor of CR rate in stage IIB cervical cancer (55.7% vs. 67.2%; adjusted OR = 2.111; 95% CI, 1.117–3.989; *P* = 0.021). As shown in Table 7, univariate and binary logistic regression analyses for the association of the inflammatory indexes with CR rate showed that NLR ≥ 2.49 was associated with decreased CR rate (58.5% vs. 71.3%; crude OR = 1.767; 95% CI, 1.054–2.963; *P* = 0.031). When adjusted by tumor size, NLR ≥ 2.49 just showed a trend to decrease CR rate (adjusted OR = 1.547; 95% CI, 0.909–2.631; *P* = 0.108). PLR, MLR, BLR, and SIRI had no correlation with CR rate in stage IIB cervical cancer.

## 4. Discussion

There were more and more studies indicating that inflammatory markers play critical roles in the prognosis of various malignant tumors [49]. Inflammatory cells release inflammatory factors and induce inflammatory response, with involvement of a variety of inflammatory mediators. Some cancers arise from inflammation, and inflammatory cells are implicated in the viability and proliferation of tumor cells by orchestrating the tumor microenvironment. Moreover, tumor cells coopt some inflammatory signaling molecules and receptors to interfere tumor progression [50]. Several inflammatory biomarkers were identified to be predictors for clinical cancer behavior involving progression and prognosis of cancers [51]. Moreover, inflammatory response triggered by tumors leads to changes in blood components, including platelets, neutrophils, monocytes, and lymphocytes [52, 53]. Unlike molecular diagnosis, PLR, NLR, MLR, BLR, and SIRI are easily obtained from blood routine examinations, which are inexpensive in price. Briefly, inflammatory indexes including PLR, NLR, MLR, BLR, and SIRI have quite a few advantages, such as convenience, low cost, simplicity, and operability. With respect to prognostic value, the inflammatory indexes were increasingly recognized to serve as prognostic markers in cancers [21–29]. Likewise, the indexes were confirmed to have prognostic value in CC patients [32–39].

There were scarce studies focusing on the patients with stage IIb CC. Only these patients were selected as the subjects of the study on the basis of the following considerations: firstly, it was controversial to explicitly distinguish between early-stage CC and advanced stage CC, and there was no unified definition of advanced stage CC worldwide. Commonly, stage IIb CC was classified as an advanced stage CC [35]; occasionally, stage IIb CC was also regarded as an early-stage CC [17]. The gap in prognosis between early and advanced cancer is large [35], stage IIb CC falls in between early-stage CC and advanced stage CC, and its prognosis is elusive as yet, and the present study contributes to explore the prognosis of patients with stage IIb CC. Secondly, subgroup analyses seek to verify the prognosis value of PLR, NLR, MLR, BLR, and SIRI in specific stage CC. In the current study, PLR exhibited no significant effect on OS in stage IIb CC. In fact, whether PLR serves as a prognostic factor in CC remains controversial. Chao et al. [39] demonstrated that elevated PLR was associated with reduced OS in early-stage CC, whereas Wang et al. [43] found no significant association between PLR and OS in CC patients containing a large proportion of early-stage CC patients. A retrospective study performed by Lee et al. [54] revealed that PLR was not a significant prognostic factor for OS. Importantly, stage IIb CC patients accounted for a large proportion of all CC patients in the hospital. Noteworthily, the FIGO stage classification used in the study was in accordance with FIGO 2009 cervical cancer staging criteria [55], instead of FIGO 2018 version. Lymphatic metastasis was an independent prognostic factor in CC; hence, stage IIb CC patients with lymphatic metastasis were classified as “stage IIc” in FIGO 2018 version [56].

In the current study, we illustrated that NLR was an independent prognostic factor as well as a risk factor for recurrence in patients with stage IIb CC. Neutrophils are involved in immunomodulation, and lymphocytes are vital immune cells modulating inflammatory response. In fact, neutrophils inhibit the activity of lymphocytes to reduce immune function, resulting in tumor progression and metastasis [57, 58]. Both neutrophils and lymphocytes are indicators of systemic inflammation [59]. Obviously, increased NLR indicates a decrease in immune function, which facilitates tumor development. As shown in our results, increased NLR was associated with larger tumor size as well as lymphatic metastasis. The results were consistent with the preceding inference. Additionally, we observed that increases in MLR during treatment were associated with worse PFS and OS. The cause for this association is unclear, but given that the correlation is specific to MLR, they may reflect elevated levels of physiologic stress. Decreasing monocyte counts may indicate hematologic toxicity, reflecting poor toleration of therapy, which in turn may lead to worse outcomes [60]. Furthermore, the ROC curves suggested that NLR and MLR had excellent predictive value in the prognosis and recurrence risk for the patients. In summary, the applications of the NLR and MLR as predictors in clinical outcome of stage IIb CC patients were promising.

Recently, it has been reported that SIRI was an independent prognostic factor in CC patients and even had better prognostic value compared to NLR, PLR, and MLR [39].

Whereas, in the current study, increased SIRI trended toward shorter OS (*P*=0.113) in the univariate Cox regression analysis and just showed a trend to reduce PFS after adjustment with lymphatic metastasis in the multivariate Cox regression analysis, our results seem to be inconsistent with those of the previous study [39]; maybe due to different analyzed cohorts, the cohort in the previous study included patients with early-stage CC, whereas our cohort focused on patients with stage IIB CC.

There were some limitations in our study. For instance, tumor invasion depth was identified to be an independently predictive factor for OS in CC patients and was generally used as an important covariate in the multivariate Cox regression analysis [39, 61]. In our cohort, the patients with stage IIB CC were advised to receive neoadjuvant chemotherapy rather than surgery, so it was inconvenient and difficult to evaluate the tumor invasion depth. Furthermore, the values of the inflammatory indexes were all cut off by corresponding median values, instead of optimal cut-off values obtained from ROC curves in accordance with Youden index [62]. Consequently, several *P* values were on the verge of 0.05 and were not statistically significant. Certainly, the practical significance of the inflammatory indexes would not vary with research methods. Due to extremely limited funds, there were several inherent limitations in the present study, including the missing of a validation cohort for the inflammatory indexes; susceptibility to bias in data selection and analysis due to the retrospective nature of the study; little contribution to the generalization of the results, because the study was conducted at a single institution.

## 5. Conclusions

In conclusion, both NLR and PLR independently predicted poor prognosis in patients with stage IIB cervical cancer. Moreover, higher NLR and MLR were associated with increased cancer recurrence risk, and NLR showed a potential use for predicting therapeutic response. Hence, the application of the inflammatory indexes in predicting clinical outcome of patients with stage IIB cervical cancer deserves popularization in light of a convenient and low-cost access to the data.

## Figures and Tables

**Table 1:** The baseline characteristics of 260 patients with stage IIB cervical cancer.

Characteristics	Totality, *n* (%)	Median (range)
Age at diagnosis	260 (100.0%)	51 (28–74) years
Menopause		
Yes	137 (52.7%)	
No	123 (47.3%)	
Age at menopause	133 (51.2%)^a^	49 (24–57) years
Pathology		
PDSCC	8 (3.1%)	
MDSCC	231 (88.8%)	
WDSCC	6 (2.3%)	
Adenocarcinoma	11 (4.2%)	
Adenosquamous carcinoma	3 (1.2%)	
Indefinite	1 (0.4%)	
Tumor size		
<4 cm	78 (30.0%)	
≥4 cm	182 (70.0%)	
Lymphatic metastasis		
Yes	61 (23.5%)	
No	196 (75.4%)	
Not evaluated	3 (1.2%)	
WBC	260 (100.0%)	6.45 (2.17–18.61) (×10^9^/L)
Neutrophil	260 (100.0%)	4.07 (0.96–16.57) (×10^9^/L)
Lymphocyte	260 (100.0%)	1.60 (0.55–3.40) (×10^9^/L)
Monocyte	260 (100.0%)	0.42 (0.04–1.08) (×10^9^/L)
RBC	260 (100.0%)	4.17 (1.58–6.57) (×10^12^/L)
Hb	260 (100.0%)	123.5 (22.0–156.0) (g/L)
Platelet	260 (100.0%)	248 (80–500) (×10^9^/L)
Basophil	260 (100.0%)	0.03 (0.01–0.17) (×10^9^/L)
PLR	260 (100.0%)	154.17 (48.48–500.00)
NLR	260 (100.0%)	2.49 (0.93–14.79)
MLR	260 (100.0%)	0.26 (0.04–1.42)
BLR	260 (100.0%)	0.021 (0.004–0.118)
SIRI	260 (100.0%)	1.02 (0.04–15.39)
Neoadjuvant chemotherapy		
Yes	243 (93.5%)	
No	17 (6.5%)	
Radiotherapy		
Yes	256 (98.5%)	
No	4 (1.5%)	
Radiotherapy dose	256 (98.5%)	56.35 (25.8–65.35) (gy)
Radiotherapy duration	256 (98.5%)	54 (10–96) (days)
Response		
CR	168 (64.6%)	
PR	82 (31.5%)	
SD	7 (2.7%)	
PD	2 (0.8%)	
Not evaluated	1 (0.4%)	
Recurrence		
Yes	70 (26.9%)	
No	190 (73.1%)	
Death		
Yes	75 (28.8%)	
No	178 (68.5%)	
Loss to follow-up	7 (2.7%)	

PDSCC, poorly differentiated squamous cell carcinoma; MDSCC, moderately differentiated squamous cell carcinoma; WDSCC, well differentiated squamous cell carcinoma; WBC, white blood cell; RBC, red blood corpuscle; Hb, hemoglobin; PLR, platelet-to-lymphocyte ratio; NLR, neutrophil-to-lymphocyte ratio; MLR, monocyte-to-lymphocyte ratio; BLR, basophil-to-lymphocyte ratio; SIRI, systemic inflammation response index; CR, complete response; PR, partial response; SD, stable disease; PD, progressive disease. ^a^The other four patients were not sure about the age at menopause.

**Table 2:** The association of inflammatory indexes with clinical characteristics in patients with stage IIB cervical cancer.

Clinical characteristics	*N*	PLR^a^	NLR^a^	MLR^a^	BLR^a^	SIRI^a^
Age						
≤51 years	139	173.87 (132.56, 215.35)	2.90 (2.11, 3.95)	0.29 (0.21, 0.37)	0.022 (0.014, 0.033)	1.22 (0.72, 1.93)
>51 years	121	126.25 (97.70, 172.36)	2.10 (1.63, 2.80)	0.23 (0.17, 0.32)	0.021 (0.015, 0.029)	0.84 (0.55, 1.25)
*P* value		<0.001	<0.001	0.001	0.399	<0.001
Menopause						
Yes	137	126.37 (99.80, 174.56)	2.11 (1.65, 2.80)	0.23 (0.17, 0.32)	0.021 (0.015, 0.030)	0.81 (0.55, 1.30)
No	123	176.79 (137.91, 218.57)	3.06 (2.14, 4.12)	0.29 (0.22, 0.39)	0.022 (0.015, 0.031)	1.22 (0.77, 2.11)
*P* value		<0.001	<0.001	<0.001	0.532	<0.001
Pathology^b^						
SCC	245	154.17 (113.58, 205.16)	2.55 (1.92, 3.54)	0.26 (0.19, 0.35)	0.021 (0.015, 0.030)	1.04 (0.64, 1.61)
Adenocarcinoma	14	159.84 (96.14, 198.12)	1.90 (1.40, 2.75)	0.22 (0.16, 0.29)	0.029 (0.019, 0.034)	0.73 (0.32, 1.33)
*P* value		0.875	0.026	0.098	0.120	0.072
Tumor size						
<4 cm	78	124.22 (95.54, 169.07)	2.08 (1.51, 2.74)	0.23 (0.17, 0.30)	0.022 (0.014, 0.030)	0.77 (0.55, 1.22)
≥4 cm	182	167.17 (124.07, 215.05)	2.75 (2.05, 3.80)	0.27 (0.20, 0.36)	0.021 (0.015, 0.031)	1.16 (0.71, 1.78)
*P* value		<0.001	<0.001	0.013	0.736	<0.001
Lymphatic metastasis						
Yes	61	182.21 (127.89, 218.90)	2.95 (2.16, 4.05)	0.30 (0.24, 0.40)	0.021 (0.015, 0.033)	1.35 (0.90, 2.23)
No	196	152.26 (108.01, 193.80)	2.32 (1.76, 3.17)	0.24 (0.17, 0.32)	0.022 (0.015, 0.030)	0.93 (0.58, 1.39)
*P* value		0.011	0.001	<0.001	0.655	<0.001
Neoadjuvant chemotherapy						
Yes	243	155.10 (115.83, 203.14)	2.56 (1.91, 3.49)	0.26 (0.19, 0.35)	0.021 (0.015, 0.030)	1.06 (0.64, 1.59)
No	17	125.00 (97.61, 205.16)	2.09 (1.43, 2.84)	0.23 (0.16, 0.31)	0.020 (0.013, 0.034)	0.73 (0.47, 1.12)
*P* value		0.271	0.178	0.350	0.763	0.039

PLR, platelet-to-lymphocyte ratio; NLR, neutrophil-to-lymphocyte ratio; MLR, monocyte-to-lymphocyte ratio; BLR, basophil-to-lymphocyte ratio; SIRI, systemic inflammation response index; SCC, squamous cell carcinoma. ^a^The values of PLR, NLR, MLR, BLR, and SIRI were all expressed as median (first quartile, third quartile). ^b^SCC (squamous cell carcinoma) consists of PDSCC (poorly differentiated squamous cell carcinoma), MDSCC (moderately differentiated squamous cell carcinoma), and WDSCC (well differentiated squamous cell carcinoma). Adenocarcinoma includes adenocarcinoma and adenosquamous carcinoma.

**Table 3:** Cox regression analysis of clinical factors influencing overall survival of stage IIB cervical cancer patients.

Clinical factors	*N*	Mean ± SE (month)	Univariate analysis		Multivariate analysis	
			HR (95% CI)	*P* value	HR (95% CI)	*P* value
Age						
≤51 years	139	72.90 ± 3.22	1 (reference)		—	
>51 years	121	79.40 ± 3.12	0.729 (0.460–1.158)	0.181	—	—
Menopause						
No	123	71.21 ± 3.50	1 (reference)		1 (reference)	
Yes	137	79.98 ± 2.90	0.653 (0.414–1.031)	0.067	0.771 (0.484–1.228)	0.274
Pathology						
SCC	245	76.32 ± 2.33	1 (reference)		—	
Adenocarcinoma	14	56.81 ± 6.27	1.429 (0.620–3.291)	0.402	—	—
Tumor size						
<4 cm	78	84.96 ± 3.41	1 (reference)		1 (reference)	
≥4 cm	182	72.05 ± 2.83	2.130 (1.191–3.809)	0.011	1.764 (0.966–3.223)	0.065
Lymphatic metastasis						
No	196	78.56 ± 2.42	1 (reference)		1 (reference)	
Yes	61	60.66 ± 4.84	2.110 (1.310–3.400)	0.002	1.806 (1.107–2.948)	0.018
Neoadjuvant chemotherapy						
Yes	243	75.62 ± 2.35	1 (reference)		—	
No	17	61.65 ± 5.90	1.243 (0.454–3.404)	0.672	—	—

CC, cervical cancer; SE, standard error; HR, hazard ratio; CI, confidence interval; SCC, squamous cell carcinoma.

**Table 4:** Cox regression analysis of clinical factors influencing progression-free survival of stage IIB cervical cancer patients.

Clinical factors	*N*	Mean ± SE (month)	Univariate analysis		Multivariate analysis	
			HR (95% CI)	*P* value	HR (95% CI)	*P* value
Age						
≤51 years	142	68.32 ± 3.39	1 (reference)		—	
>51 years	128	70.97 ± 3.63	0.844 (0.559–1.275)	0.420	—	—
Menopause						
Yes	144	65.30 ± 3.70	1 (reference)		1 (reference)	
No	126	73.81 ± 3.25	0.687 (0.456–1.037)	0.074	0.758 (0.498–1.154)	0.197
Pathology						
SCC	255	69.73 ± 2.56	1 (reference)		—	
Adenocarcinoma	14	54.14 ± 7.14	1.130 (0.493–2.586)	0.773	—	—
Tumor size						
<4 cm	82	76.05 ± 4.16	1 (reference)		1 (reference)	
≥4 cm	188	67.00 ± 3.02	1.514 (0.938–2.445)	0.09	1.261 (0.764–2.080)	0.365
Lymphatic metastasis						
Yes	62	72.28 ± 2.72	1 (reference)		1 (reference)	
No	205	55.11 ± 4.98	1.888 (1.220–2.923)	0.004	1.736 (1.107–2.723)	0.016
Neoadjuvant chemotherapy						
Yes	250	70.07 ± 2.71	1 (reference)		—	
No	20	71.70 ± 5.84	0.907 (0.555–1.687)	0.907	—	—

CC, cervical cancer; SE, standard error; HR, hazard ratio; CI, confidence interval; SCC, squamous cell carcinoma.

**Table 5:** Cox regression analysis for predictive value of inflammatory indexes in prognosis of stage IIB cervical cancer.

Prognosis	Inflammatory indexes		*N*	Mean ± SE (month)	HR (95% CI)	*P* value	HR (95% CI)	*P* value
OS	PLR							
		<154.17	129	76.47 ± 3.14	1 (reference)		1 (reference)	
		≥154.17	131	74.64 ± 3.21	1.049 (0.667–1.649)	0.837	1.044 (0.662–1.645)	0.854
	NLR							
		<2.49	130	82.27 ± 2.81	1 (reference)		1 (reference)	
		≥2.49	130	69.02 ± 3.40	1.960 (1.227–3.131)	0.005	1.721 (1.068–2.773)	0.026
	MLR							
		<0.26	131	82.55 ± 2.76	1 (reference)		1 (reference)	
		≥0.26	129	69.17 ± 3.49	2.012 (1.258–3.217)	0.003	1.780 (1.099–2.881)	0.019
	BLR							
		<0.021	129	75.26 ± 3.25	1 (reference)		1 (reference)	
		≥0.021	131	75.77 ± 3.12	0.948 (0.603–1.492)	0.818	0.902 (0.573–1.419)	0.654
	SIRI							
		<1.02	130	79.56 ± 2.93	1 (reference)		1 (reference)	
		≥1.02	130	72.46 ± 3.38	1.446 (0.916–2.283)	0.113	1.271 (0.796–2.028)	0.315
PFS	PLR							
		<154.17	129	70.48 ± 3.44	1 (reference)		1 (reference)	
		≥154.17	131	68.48 ± 3.48	1.082 (0.719–1.629)	0.706	1.004 (0.665–1.514)	0.986
	NLR							
		<2.49	130	77.22 ± 3.15	1 (reference)		1 (reference)	
		≥2.49	130	62.18 ± 3.58	1.944 (1.274–2.967)	0.002	1.736 (1.129–2.669)	0.012
	MLR							
		<0.26	131	77.35 ± 3.14	1 (reference)		1 (reference)	
		≥0.26	129	62.33 ± 3.65	1.992 (1.303–3.043)	0.001	1.806 (1.169–2.791)	0.008
	BLR							
		<0.021	129	68.89 ± 3.51	1 (reference)		1 (reference)	
		≥0.021	131	70.37 ± 3.36	0.941 (0.625–1.417)	0.769	0.888 (0.589–1.338)	0.570
	SIRI							
		<1.02	130	74.76 ± 3.26	1 (reference)		1 (reference)	
		≥1.02	130	64.12 ± 3.81	1.538 (1.015–2.329)	0.042	1.379 (0.901–2.110)	0.139

PLR, platelet-to-lymphocyte ratio; NLR, neutrophil-to-lymphocyte ratio; MLR, monocyte-to-lymphocyte ratio; BLR, basophil-to-lymphocyte ratio; SIRI, systemic inflammation response index; OS, overall survival. PFS, progression-free survival; HR, hazard ratio; CI, confidence interval.

**Table 6:** Relationship between inflammatory indexes and recurrence in patients with stage IIB cervical cancer.

Inflammatory indexes	Recurrence (%)	OR (95% CI)	*P* value
PLR			
<154.17	25.3	1 (reference)	
≥154.17	27.8	1.103 (0.635–1.912)	0.729
NLR			
<2.49	19.8	1 (reference)	
≥2.49	30.5	1.704 (0.913–3.185)	0.046
MLR			
<0.26	19.8	1 (reference)	
≥0.26	37.2	2.392 (1.370–4.184)	0.002
BLR			
<0.021	27.7	1 (reference)	
≥0.021	28.5	1.017 (0.592–1.745)	0.952
SIRI			
<1.02	23.1	1 (reference)	
≥1.02	31.9	1.536 (0.885–2.667)	0.126

PLR, platelet-to-lymphocyte ratio; NLR, neutrophil-to-lymphocyte ratio; MLR, monocyte-to-lymphocyte ratio; BLR, basophil-to-lymphocyte ratio; SIRI, systemic inflammation response index; OR, odds ratio; CI, confidence interval.

**Table 7:** Univariate and binary logistic regression analyses for the association of inflammatory indexes with complete remission rate in patients with stage IIB cervical cancer.

Inflammatory indexes	CR, *n* (%)	Univariate analysis		Binary logistic regression analysis	
		OR (95% CI)	*P* value	OR (95% CI)	*P* value
PLR					
<154.17	87 (68.0%)	1 (reference)		1 (reference)	
≥154.17	81 (61.8%)	1.310 (0.785–2.186)	0.301	1.093 (0.641–1.863)	0.745
NLR					
<2.49	92 (71.3%)	1 (reference)		1 (reference)	
≥2.49	76 (58.5%)	1.767 (1.054–2.963)	0.031	1.547 (0.909–2.631)	0.108
MLR					
<0.26	88 (67.2%)	1 (reference)		1 (reference)	
≥0.26	80 (62.5%)	1.228 (0.737–2.047)	0.431	1.129 (0.670–1.902)	0.648
BLR					
<0.021	80 (62.0%)	1 (reference)		1 (reference)	
≥0.021	88 (67.7%)	0.779 (0.467–1.299)	0.339	0.807 (0.480–1.357)	0.419
SIRI					
<1.02	82 (63.1%)	1 (reference)		1 (reference)	
≥1.02	86 (66.7%)	0.854 (0.513–1.424)	0.545	0.702 (0.411–1.198)	0.194

PLR, platelet-to-lymphocyte ratio; NLR, neutrophil-to-lymphocyte ratio; MLR, monocyte-to-lymphocyte ratio; BLR, basophil-to-lymphocyte ratio; SIRI, systemic inflammation response index; CR, complete remission; OR, odds ratio; CI, confidence interval.

## Data Availability

The datasets used and/or analyzed during the current study are available from the corresponding author on reasonable request.

## Supplementary Materials

Supplementary Materials Supplementary Table S1: chi-square test for the evaluation of relevance between tumor size and lymphatic metastasis. Supplementary Table S2: relationship between clinical factors and recurrence in patients with stage IIB cervical cancer. Supplementary Table S3: univariate and binary logistic regression analyses for the association of clinical factors with CR rate in patients with stage IIB cervical cancer.
