# Supplementary material for: Neutrophil-to-Lymphocyte Ratio (NLR) and Monocyte-to-Lymphocyte Ratio (MLR) Predict Clinical Outcome in Patients with Stage IIB Cervical Cancer
Source: J Oncol. 2021 Sep 8;2021:2939162. doi: 10.1155/2021/2939162 (PMC8443385; doi:10.1155/2021/2939162)
Supplement: Supplementary Materials — Supplementary Table S1: chi-square test for the evaluation of relevance between tumor size and lymphatic metastasis. Supplementary Table S2: relationship between clinical factors and recurrence in patients with stage IIB cervical cancer. Supplementary Table S3: univariate and binary logistic regression analyses for the association of clinical factors with CR rate in patients with stage IIB cervical cancer. [file 2939162.f1.zip › Table S1.docx]

**Table S1. Chi-square test for the evaluation of relevance between tumor size and lymphatic metastasis.**

|  |  | lymphatic metastasis, n (%) | | OR (95% CI) | P value |
| --- | --- | --- | --- | --- | --- |
|  |  | Yes | No |  |  |
| Tumor size | < 4 cm | 8 (10.4%) | 69 (89.6%) | 1 (reference) |  |
|  | ≥ 4 cm | 53 (29.4%) | 127 (70.6%) | 3.60 (1.62 – 8.00) | 0.001 |

Abbreviations: OR, odds ratio; CI, confidence interval.
