# Supplementary material for: Neutrophil-to-Lymphocyte Ratio (NLR) and Monocyte-to-Lymphocyte Ratio (MLR) Predict Clinical Outcome in Patients with Stage IIB Cervical Cancer
Source: J Oncol. 2021 Sep 8;2021:2939162. doi: 10.1155/2021/2939162 (PMC8443385; doi:10.1155/2021/2939162)
Supplement: Supplementary Materials — Supplementary Table S1: chi-square test for the evaluation of relevance between tumor size and lymphatic metastasis. Supplementary Table S2: relationship between clinical factors and recurrence in patients with stage IIB cervical cancer. Supplementary Table S3: univariate and binary logistic regression analyses for the association of clinical factors with CR rate in patients with stage IIB cervical cancer. [file 2939162.f1.zip › Table S2.docx]

**Table S2. Relationship between clinical factors and recurrence in patients with stage ⅡB cervical cancer.**

| **Clinical factors** | **Recurrence (%)** | **OR (95% CI)** | **P value** |
| --- | --- | --- | --- |
| Age |  |  |  |
| ≤ 51 years | 28.2 | 1 (reference) |  |
| > 51years | 23.5 | 0.772 (0.440 – 1.353) | 0.366 |
| Menopause |  |  |  |
| No | 29.7 | 1 (reference) |  |
| Yes | 23.8 | 0.737 (0.426 – 1.277) | 0.277 |
| Pathology |  |  |  |
| SCC | 27.5 | 1 (reference) |  |
| Adenocarcinoma | 37.1 | 1.475 (0.477 – 4.566) | 0.497 |
| Tumor size |  |  |  |
| < 4 cm | 24.3 | 1 (reference) |  |
| ≥ 4 cm | 29.6 | 1.311 (0.714 – 2.404) | 0.382 |
| Lymphatic metastasis |  |  |  |
| No | 25.2 | 1 (reference) |  |
| Yes | 39.2 | 1.946 (1.060 – 3.571) | 0.030 |
| Neoadjuvant chemotherapy |  |  |  |
| Yes | 27.7 | 1 (reference) |  |
| No | 30.3 | 1.094 (0.371 – 3.226) | 0.870 |

**Abbreviations:** OR, odds ratio; hazard ratio; CI, confidence interval; SCC, squamous cell carcinoma.
