# Supplementary material for: Neutrophil-to-Lymphocyte Ratio (NLR) and Monocyte-to-Lymphocyte Ratio (MLR) Predict Clinical Outcome in Patients with Stage IIB Cervical Cancer
Source: J Oncol. 2021 Sep 8;2021:2939162. doi: 10.1155/2021/2939162 (PMC8443385; doi:10.1155/2021/2939162)
Supplement: Supplementary Materials — Supplementary Table S1: chi-square test for the evaluation of relevance between tumor size and lymphatic metastasis. Supplementary Table S2: relationship between clinical factors and recurrence in patients with stage IIB cervical cancer. Supplementary Table S3: univariate and binary logistic regression analyses for the association of clinical factors with CR rate in patients with stage IIB cervical cancer. [file 2939162.f1.zip › Table S3 (1).docx]

**Table S3. Univariate and binary logistic regression analyses for the association of clinical factors with CR rate in patients with stage ⅡB cervical cancer.**

| **Clinical factors** | **CR, n (%)** | **Univariate analysis** | |  | **Binary logistic regression analysis** | |
| --- | --- | --- | --- | --- | --- | --- |
|  |  | **OR (95% CI)** | **P value** |  | **OR (95% CI)** | **P value** |
| Age |  |  |  |  |  |  |
| ≤ 51 years | 83 (59.7%) | 1 (reference) |  |  | 1 (reference) |  |
| > 51years | 85 (70.8%) | 0.610 (0.363 – 1.026) | 0.062 |  | 0.812 (0.374 – 1.763) | 0.598 |
| Menopause |  |  |  |  |  |  |
| No | 72 (58.5%) | 1 (reference) |  |  | 1 (reference) |  |
| Yes | 96 (70.6%) | 0.798 (0.461 – 1.381) | 0.042 |  | 0.804 (0.372 – 1.737) | 0.579 |
| Pathology |  |  |  |  |  |  |
| SCC | 160 (65.6%) | 1 (reference) |  |  | _ |  |
| Adenocarcinoma | 7 (50.0%) | 1.905 (0.646 – 5.618) | 0.236 |  | _ | _ |
| Tumor size |  |  |  |  |  |  |
| < 4 cm | 60 (77.9%) | 1 (reference) |  |  | 1 (reference) |  |
| ≥ 4 cm | 108 (59.3%) | 2.415 (1.309 – 4.464) | 0.004 |  | 2.111 (1.117 – 3.989) | 0.021 |
| Lymphatic metastasis |  |  |  |  |  |  |
| No | 131 (67.2%) | 1 (reference) |  |  | 1 (reference) |  |
| Yes | 34 (55.7%) | 1.626 (0.903 – 2.924) | 0.103 |  | 1.328 (0.724 – 2.437) | 0.359 |
| Neoadjuvant chemotherapy |  |  |  |  |  |  |
| Yes | 156 (64.5%) | 1 (reference) |  |  | _ |  |
| No | 12 (70.6%) | 0.756 (0.258 – 2.217) | 0.609 |  | _ | _ |

**Abbreviations:** CR, complete remission; OR, odds ratio; CI, confidence interval; SCC, squamous cell carcinoma.
